# HIV Drug Resistance Surveillance Using Pooled Pyrosequencing

**DOI:** 10.1371/journal.pone.0009263

**Published:** 2010-02-17

**Authors:** Hezhao Ji, Nathalie Massé, Shaun Tyler, Ben Liang, Yang Li, Harriet Merks, Morag Graham, Paul Sandstrom, James Brooks

**Affiliations:** 1 National HIV and Retrovirology Laboratories, National Microbiology Laboratory, Public Health Agency of Canada, Ottawa, Canada; 2 Genomics Core Facility, National Microbiology Laboratory, Public Health Agency of Canada, Winnipeg, Canada; 3 Department of Medical Microbiology, University of Manitoba, Winnipeg, Canada; Tsinghua University, China

## Abstract

**Background:**

Surveillance for HIV transmitted drug resistance (TDR) is performed using HIV genotype results from individual specimens. Pyrosequencing, through its massive parallel sequencing ability, can analyze large numbers of specimens simultaneously. Instead of using pyrosequencing conventionally, to sequence a population of viruses within an individual, we interrogated a single combined pool of surveillance specimens to demonstrate that it is possible to determine TDR rates in HIV protease from a population of individuals.

**Methodology/Principal Findings:**

The protease region from 96 treatment naïve, HIV+ serum specimens was genotyped using standard Sanger sequencing method. The 462 bp protease amplicons from these specimens were pooled in equimolar concentrations and re-sequenced using the GS FLX Titanium system. The nucleotide (NT) and amino acid (AA) differences from the reference sequence, along with TDR mutations, detected by each method were compared. In the protease sequence, there were 212 nucleotide and 81 AA differences found using conventional sequencing and 345 nucleotide and 168 AA differences using pyrosequencing. All nucleotide and amino acid polymorphisms found at frequencies ≥5% in pyrosequencing were detected using both methods with the rates of variation highly correlated. Using Sanger sequencing, two TDR mutations, M46L and I84V, were each detected as mixtures at a frequency of 1.04% (1/96). These same TDR mutations were detected by pyrosequencing with a prevalence of 0.29% and 0.34% respectively. Phylogenetic analysis established that the detected low frequency mutations arose from the same single specimens that were found to contain TDR mutations by Sanger sequencing. Multiple clinical protease DR mutations present at higher frequencies were concordantly identified using both methods.

**Conclusions/Significance:**

We show that pyrosequencing pooled surveillance specimens can cost-competitively detect protease TDR mutations when compared with conventional methods. With few modifications, the method described here can be used to determine population rates of TDR in both protease and reverse transcriptase. Furthermore, this pooled pyrosequencing technique may be generalizable to other infectious agents where a survey of DR rates is required.

## Introduction

Surveillance of transmitted HIV drug resistance (TDR) is an essential public health component of a comprehensive HIV strategy. Information obtained from TDR surveillance facilitates individual drug selection, where there are many available combinations, and informs drug selection for national treatment programs in resource-limited settings[1]–[3]. TDR surveillance may be performed through comprehensive surveillance or through structured sampling methods [4]. Independent of the approach, TDR surveillance relies upon individual genotyping of surveillance specimens using Sanger sequencing. Drawbacks intrinsic to conventional Sanger sequencing are the variable subjective interpretations of sequencing results, limited sensitivity for minor sequence variants, and cost. Any technique that removes subjective interpretation, enhances sensitivity, and has the potential to lower costs would assist with global HIV TDR surveillance.

Pyrosequencing provides massive parallel sequencing that can be used to produce complete genome coverage from a conserved sequence or create an array of reads from mixed sequences[5], [6]. Among heterogeneous collections of sequences, the current applications of pyrosequencing have been to, either resolve the sequences from different organisms in the sample[7], [8] or be used to probe extremely low frequency variants existing within a single target[9]–[12]. Within HIV genetics, there has been a great deal of interest in the application of pyrosequencing to address questions oriented around the genetic diversity of the virus within an individual. Instead of using the strengths of pyrosequencing to sequence a population of viruses from within an individual, we exploited the technique to determine the sequences of viruses from within a population of individuals. As a proof of concept that this approach can be used to determine the prevalence of surveillance TDR mutations, we pyrosequenced an equimolar pool of HIV protease amplicons from a drug naïve HIV infected population and determined rates of protease TDR.

## Materials and Methods

### Subjects and Specimens

Ninety-six anonymized, remnant serum specimens from an IRB approved national HIV TDR surveillance program were used in the analysis. Previous work had shown that the specimen subtypes were 90 subtype B; 4 subtype C, and one each of A1 and CRF02_AG. HIV-1 subtypes were determined using REGA HIV-1 Automated Subtyping Tool based upon *pol* sequences (http://www.bioafrica.net/virus-genotype/html/subtyping.html).

### Bulk Sanger Sequencing-Based HIV-1 DR Genotypic Test

HIV-1 nucleic acid was extracted from 200 µl of serum using the Nuclisens EasyMag system (Biomerieux, Canada) following manufacturer's instructions. HIV-1 protease (PR) and reverse transcriptase (RT), up to codon 236, were bidirectional sequenced with an in-house protocol. Briefly, viral RNA was reverse transcribed and amplified according to the manufacturer's directions using the QIAGEN one-step RT-PCR kit (QIAGEN, Canada). RT-primers were POF 5′-TGAARGAITGYACTGARAGRCAG GCTAAT-3′ and POR 5′-CCTCATTYTTGCATAYTTYCCTGTT-3′ with nested primers PIF 5′- YTCAGARCAGRCCRGARCCAACAGC-3′ and PIR 5′-GGYTCTTGRTAAATTTGRTATGTCCA-3′. All reactions were carried out using standard conditions with annealing temperatures of 53°C. PCR amplicons were then purified and diluted to 15 ng/µl for DNA sequencing using ABI Prism BigDye 3.1 Cycle Sequencing System (Applied Biosystems, USA) following manufacturer's instructions. The sequencing PCR primers included both nested PCR primers and two additional primers PS1 5′-CTGGTGTYTCATTRTTKRTACTAGGT-3′ and PS2 5′-TTYTGGGARGTYCARY TAGGRATACC-3′.

### Pyrosequencing

Briefly, a 462 nucleotide fragment containing the entire PR gene was produced by amplifying RT-PCR products using PIF-p 5′-TCCCTCARATCACTCTTTGG-3′ and PIR-p 5′-GGRTTTTYAGGCCCAATTTT-3′ with an annealing temperature of 59°C. Individual PCR products were purified using Millipore's Amicon Microcon-PCR Centrifugal filter devices (Millipore, USA), pooled in equimolar amount with Beckman Biomex FX system equipped with a DTX880 Multimode Detector (Beckman, USA), and sequenced using the GS FLX Titanium pyrosequencing kit (Roche Applied Science. USA). Pyrosequencing of the 96 specimens was conducted using 1/16 the capacity of a full PicoTiterPlate. Overall the reaction produced 42,928 sequence reads. Further analysis was only performed on the 19,106 (44.5%) reads that were: passed by the quality control software; of sufficient read length to cover the amplicon and; successfully mapped to the HXB-2 reference sequence. Based on the approximation of the expected pyrosequencing read length (400∼500 bp) and our template size (462 bp), the mapping criteria was 60% overlapping and 75% matching to the reference.

The frequency of differences from HIV-1 HXB2 (Accession number: K03455) was calculated for all pyrosequencing reads. The polymorphism and DR rates obtained from the pooled pyrosequencing reads were then compared with the polymorphism and DR rates calculated from the Sanger sequencing reads. Initial comparisons were done for all detected polymorphisms including those present at an extremely low levels. Analysis was repeated on those polymorphisms that were present at frequencies ≥0.2%. This threshold was chosen to be comparable with the sensitivity of Sanger sequencing based on the following assumptions: under ideal circumstances bulk, Sanger sequencing can detect a 20% mixture; the pool of specimens will consist of 100 specimens; thus, a 20% mixture in a single specimen will be present in the 96-member pyrosequencing pool at 0.2%.

### Sequence Analysis and HIV-1 DR Mutation Determination

Conventional sequences were assembled and edited in Seqscape v2.5 (Applied Biosystems, USA) with sequence variations identified by aligning the sequences with HXB-2 (Accession number: K03455). All polymorphisms/mutations were confirmed through agreement of bidirectional sequencing results. The 19,106 valid pyrosequencing reads were aligned with HXB-2 and the frequency and distribution of nucleotide and AA changes determined using an in-house Perl script. TDR mutations were identified using a surveillance drug resistance mutation (SDRM) list [3] with additional clinical protease mutations identified according to the IAS-USA HIV DR mutation list [13]. The prevalence of polymorphisms, TDR and clinical mutations in HIV PR region were identified using conventional and pyrosequencing methods and the results compared.

### Phylogenetic Analysis

Phylogenetic analysis was performed on the group of pyrosequencing reads, containing the TDR mutation of interest, in a background of the 96 sequences obtained from Sanger sequencing. Trees were constructed using Neighbor-Joining approach using the K-2-P model with 100 bootstrap replicates as implemented in MEGA 4.0[14].

## Results

### Concordance of the Detection of NT and AA Variations Is Proportional to Their Frequency

Using conventional Sanger sequencing-based genotypic DR test satisfactory results were obtained from all 96 specimens. Among all of the genotypes, a total of 212 nucleotide (NT) and 81 amino acid (AA) differences from HXB-2 were identified in the PR region. The 19,106 satisfactory pyrosequencing reads (average length: 437) represented an average redundancy of 17,911 for each nucleotide locus. Statistically, each base position of every constituent specimen in the pooled mixture was oversampled 187 times. Analysis of all of the reads demonstrated an aggregate of 1173 NT and 995 AA differences from HXB2. Re-analysis of the data, identifying only those variations present at a level of ≥0.2 revealed 345 NT and 168 AA differences from the reference. The numbers of sequence variations detected and categorized according to the frequency of detection by pyrosequencing are shown in **Table 1**. All NT and AA changes detected by Sanger sequencing were identified by pyrosequencing with the latter method showing a greater ability to detect low abundance variants.

**Table 1:** Sequence variations detected by pyrosequencing and Sanger sequencing- based genotypic DR test.

Variation Frequency (%) a	Nucleotide Variations			Amino acid variations		
	Pyrosequencing	Sanger sequencing b	Δ % (ABS±SD)^c^	Pyrosequencing	Sanger sequencing	Δ % (ABS±SD)^c^
0.2∼0.49	132	17	0.7±0.1	81	9	0.7±0.1
0.5∼0.99	40	24	0.3±0.3	14	2	0.3±0.0
1∼4.99	87	84	0.8±0.8	37	29	0.8±1.0
5∼9.99	47	47	1.6±1.8	21	21	1.8±1.6
10∼14.99	14	14	2.3±1.6	4	4	3.0±2.2
15∼19.99	6	6	1.8±1.9	1	1	3.78
20∼29.99	9	9	5.3±6.1	3	3	2.0±2.6
> = 30%	10	10	6.1±10.4	7	7	2.2±1.5

*^a^* *: The categorization of frequencies based on the frequencies of variations identified by pyrosequencing;*

*^b^* *Number of nucleotide and amino acid variation listed under Sanger sequencing is the sum of all variants identified in the Sanger sequences that were detected by pyrosequencing at the given frequency;*

*^c^* *: Absolute value of the difference in variation frequency of nucleotide (NT) or amino acid (AA) frequency between the two approaches for concordantly detected mutations (Mean± standard deviation).*

**Figure 1:**
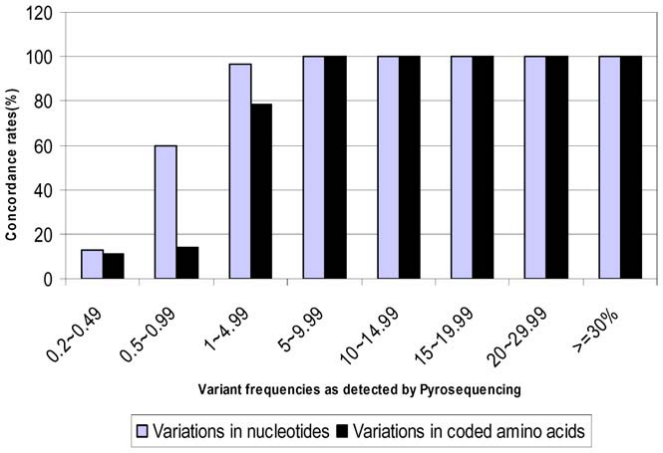
Concordance of variations detection by pyrosequencing and Sanger sequencing. The concordance rates were calculated as percentage of pyrosequencing detected sequence variations that were also observed in Sanger sequencing. Frequency ranges are categorized based on those detected by pyrosequencing.

The concordance rates between methods, for detecting NT and AA changes, were proportional to their abundances in the viral population (**Figure 1**). All NT changes present at levels of ≥5% were identified by both methods with Sanger sequencing identifying 96.6% and 24% of the polymorphisms identified by pyrosequencing at the 1∼5%, and 0.2∼1% levels respectively. Similarly, the concordance rate for AA changes was 100% for those variations present at levels of ≥5%, 78.4% for those at 1∼5%, and 11.6% for those detected in less than 1% of the pyrosequencing reads. Discrepancies in the frequency of identified variations between methods occurred exclusively among the low frequency variants detected by pyrosequencing. Furthermore, these low frequency variants represented the majority of differences from the reference with 92.7% of NT and 96.4% of AA changes present at less than 5% and 85.3% of NT and 92.7% of AA variations occurring in less than 1% of the reads.

### Proportional Representation of Detected NT and AA Variants

Comparison of the measured frequency of variants by pyrosequencing, or the calculated frequency of variants by Sanger sequencing, showed that both methods produced comparable results across a range of frequencies (**Table 1**). The greatest discrepancies between the two methods were identified at the extreme frequency ranges (<1% or ≥30%) and where variants were found at the termini of pyrosequenced fragments.

### Detection of HIV Protease DR Mutations

**Figure 2:**
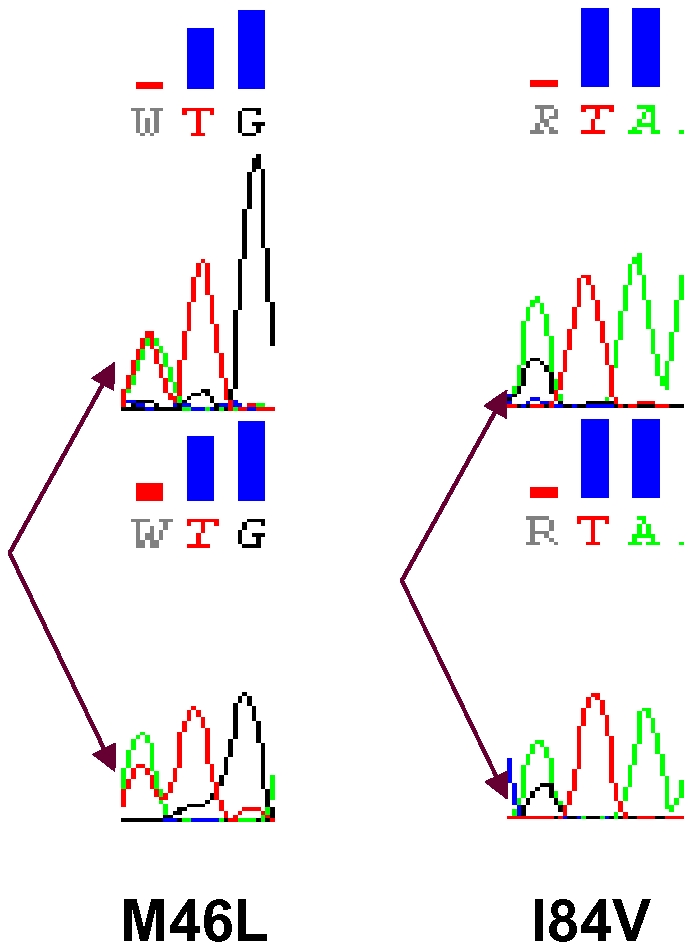
Bidirectional sequence electropherograms for the two TDR mutations detected by conventional Sanger sequencing. Two TDR mutations, M46L and I84V, were detected by conventional Sanger sequencing, each in one of the 96 examined specimens. Electropherograms demonstrate that both mutations existed as a component of a mixture.

**Figure 3:**
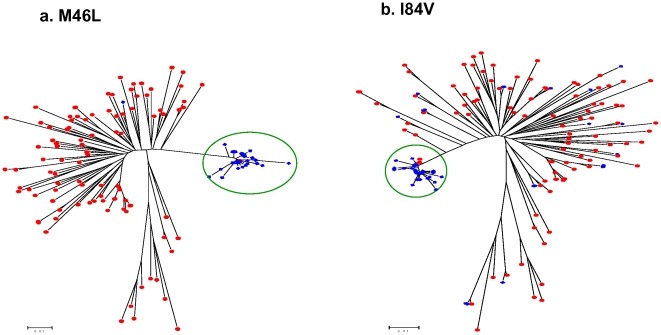
Phylogenetic analysis on pyrosequencing reads with SDRMs detected in bulk, Sanger sequencing. All positive pyrosequencing reads containing M46L (a) and I84V (b) were analyzed with Sanger sequences from the 96 specimens using Neighbour-Joining (K-2-P) with 100 bootstraps. The pyrosequencing reads with corresponding SDRM are shown in blue with the 96 Sanger sequences shown in red.

**Figure 4:**
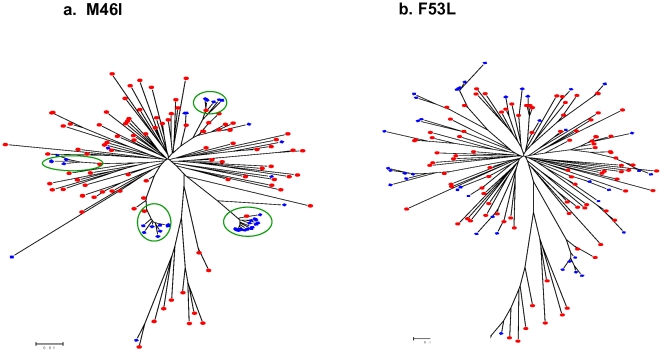
Phylogenetic analysis of pyrosequencing reads with SDRMs not detected by bulk Sanger sequencing. All positive pyrosequencing reads containing M46I (a) and F53L (b) were analyzed with Sanger sequences from the 96 specimens using Neighbour-Joining (K-2-P) with 100 bootstraps. The pyrosequencing reads with corresponding SDRM are shown in blue with the 96 Sanger sequences shown in red. Clusters of pyrosequencing reads are indicated with a green circle.

Thirteen protease TDR mutations were detected by pyrosequencing at frequencies ranging from 0.20% to 0.64% (**Table 2**). Two of these TDR mutations: M46L and I84V, detected at frequencies of 0.29% and 0.34% respectively, were also found in two Sanger sequences as mixed base calls (**Figure 2**). We performed a phylogenetic analysis using pyrosequencing reads containing individual TDR mutations in a background of the 96 Sanger sequences. The M46L and I84V mutations overwhelmingly clustered with the appropriate Sanger sequences that had the mixtures identified on the electropherograms (**Figure 3**). Among those TDR detected by pyrosequencing but not detected by Sanger sequencing, two distinct patterns emerged. In the first case, there was a clear association of pyrosequencing reads with single Sanger sequences; however, the absolute number of associated reads was much lower than in situations in which corresponding mutations were seen in the Sanger reads (**Figure 4a**). In the second case, the phylogenetic analysis did not demonstrate any significant association of the pyrosequencing reads with any single Sanger sequences (**Figure 4b**). These results are representative of all examined DR mutations as well as those variations at non-DRM sites detected by pyrosequencing only (Data Not Shown).

**Table 2:** HIV-1 protease DRMs identified by pyrosequencing and Sanger sequencing.

Protease TDR Mutations(WHO SDRM)			Minor Protease DRMs(IAS-USA 2008)		
Mutations	%(Sanger)	%(Pyro)	Mutations	%(Sanger)	%(Pyro)
M46L	1.04	0.29	L10V	4.17	5.03
I84V	1.04	0.34	G16E	6.25	4.95
M46I	0	0.35	K20R	0	0.64
D30N	0	0.22	K20I	2.08	1.74
I47V	0	0.27	L33I	2.08	2.31
I50V	0	0.58	L33V	6.25	7.42
F53L	0	0.64	M36I	19.79	14.06
I54T	0	0.24	D60E	10.42	10.84
G73S	0	0.20	I62V	22.92	23.07
V82A	0	0.47	I64M	1.04	1.22
I85V	0	0.25	I64L	5.21	6.30
N88D	0	0.35	I64V	11.46	7.60
N88S	0	0.48	H69K	11.46	9.66
			A71T	10.42	6.63
			A71V	8.33	10.44
			V77I	33.33	35.78
			V82I	1.04	1.46
			I93L	62.50	62.43

**Figure 5:**
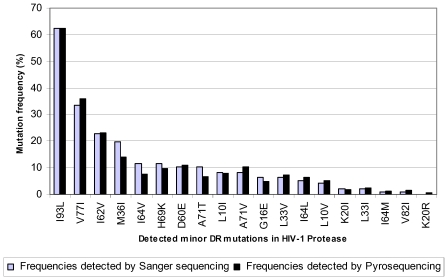
Consistent and comparable frequency readouts for minor protease DRMs by the two approaches. Eighteen minor DR mutations (IAS-USA 2008) were detected by either pyrosequencing or Sanger sequencing among the 96 specimens. Individual mutations are plotted against thee frequency detected by each Chart shows the frequency at which the individual mutations were detected by each method.

**Figure 6:**
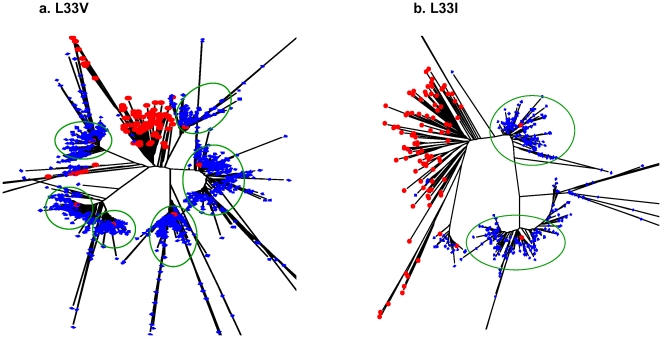
Phylogenetic analysis on pyrosequencing reads with minor DRMs and Sanger sequences from all 96 pooled specimens. All positive pyrosequencing reads containing L33V (a) and L33I (b) were analyzed with Sanger sequences from the 96 specimens using Neighbour-Joining (K-2-P) with 100 bootstraps. The pyrosequencing reads with corresponding SDRM are shown in blue with the 96 Sanger sequences shown in red. Clusters of pyrosequencing reads are indicated with a green circle.

Except for K20R which was detected by pyrosequencing only, 17 clinical, minor protease DR mutations were detected by both methods with extremely high concordance across a broad range of rates (**Figure 5**). Phylogenetic analysis using pyrosequencing reads containing specific minor protease mutations again showed that these reads overwhelmingly clustered with the correct number of Sanger sequences containing the concordant mutations. There are six clusters of pyrosequencing reads that cluster around the six L33V mutant sequences detected by Sanger sequencing (**Figure 6a**). The L33I was detected in only two Sanger reads and correspondingly the majority of the pyrosequencing reads containing the same mutation cluster around those two sequences (**Figure 6b**).

## Discussion

Successful TDR surveillance programs typically acquire sequencing results from large numbers of antiretroviral naïve subjects and produce an estimate of the percentage of drug resistance based upon the aggregate results. The percentage resistance to protease inhibitors is not described in the context of the individual but instead is attributed to the population under study. Thus far, pyrosequencing of HIV has been used to explore HIV DR in a population of viruses within an individual [6], [9], [11], [12]. However, in this proof of concept study, we use pyrsosequencing on pooled specimens in order to survey for protease DRM contained in viruses within a population.

The findings presented here are strongly supportive of analyzing pooled specimens for the determination of the prevalence of HIV TDR in HIV PR. The concordance of DRM detection when the results from PR sequencing were analyzed is astonishing. At a mutation level detected by pyrosequencing of greater than 5%, there is a 100% concordance of the prevalence of specific NT or AA changes. The overall correlation between all protease DR mutations between methods is similarly impressive. In situations where the mutations were present at frequencies greater than 1%, phylogenetic analysis demonstrated that there were a discrete number of clusters that corresponded to the number of Sanger sequences with that mutation. This finding further demonstrates that the results from pooled pyrosequencing reflect the bulk sequence content of the constituent specimens.

Determining the protease TDR prevalence using dendograms derived from the pyrosequencing reads presented an interesting challenge. Pyrosequencing reads of both the SDR mutations, M46L and I84V, identified by Sanger sequencing, were found be tightly clustered and almost exclusively associated with the mutant Sanger sequences. These mutations were detected as 0.29% and 0.34% of the total number of reads consistent with their presence as mixtures in the bulk Sanger sequence. The dendograms for the M46L and I84V mutations detected by pyrosequencing were unique among those SDRM found at frequencies of less than 1%. With further validation, this pattern of clustering may allow one to reproducibly identify those mutations that would also be observed as mixtures in Sanger sequences. We also observed cases where there were only a few mutant pyrosequencing reads but they were related, and they also clustered with a specific Sanger sequence. We believe that the observed mutant reads reflect minority variants undetectable by bulk sequencing methodology. Finally, there were the occasional mutant pyrosequencing reads that were not clearly associated with any single sequences and these may represent PCR artifact only. Further studies will confirm the predictive value of phylogenetic patterns of pyrosequencing reads but one implication is that thresholds for calling mixtures in TDR surveillance need to be standardized.

The possibility of falsely elevating the rate of TDR by separately counting TDR mutations arising from a single specimen was addressed in this study. After reviewing 803 pyrosequencing reads with TDR mutations in PR, we found 38 reads that contained more than one drug resistance mutation (DRM) in this region. The reads with more than one mutation were not associated with any one single DRM and occurred in less than 5% of the mutant reads. Thus, the absolute contribution of a multiply drug resistant specimen to the final results is negligible.

Future studies that would allow for reporting on linked mutations that exist in one coding region, or linked mutations in different coding regions in *pol*, will require some means of associating individual pyrosequencing reads. In fact in this study, the observation of high rates of non-SDRM protease mutations is entirely consistent with single specimens harbouring multiple DRMs. Methods have been described for constructing haplotypes from pyrosequencing reads using computational methods[15]; however the ability to implement these techniques in a population of 96 different viruses is unknown. Using multiplex identifiers would not only allow for TDR surveillance of mutations within protease and reverse transcriptase but also facilitate recognition of multiple linked DRM within a single specimens[15]. Current multiplex identifier techniques for pyrosequencing allow identification of 151 separate specimens which is more than sufficient for a surveillance study on the scale of threshold survey [16], [17].

To further evaluate the application potential of pyrosequencing in HIV DR surveillance, we calculated and compared the material and labor costs for Sanger sequencing and pyrosequencing (Table S1, S2 and S3). For these calculations it was assumed that the respective instruments were embedded, as components of an institutional core facility, as would be expected for a laboratory capable of performing specialized HIV DR testing. The combined labour and material costs of HIV genotyping using our in-house Sanger sequencing method was $82/specimen which is less than half the cost of commercial genotyping. Due to the common steps involved, the cost of determining drug resistance in protease is not halved but falls to $52/specimen (Table S3). In comparison, the total cost for the equivalent analysis of protease using pyrosequencing was $32/specimen. For pooled pyrosequencing based surveillance of TDR in protease and reverse transcriptase, assuming sequencing of three overlapping regions, costs are predicted to be $53/specimen (Table S3). These calculations include costs for the additional labour required for pyrosequencing. Pyrosequencing costs may, in fact, continue to fall due to competition and the increased market penetrance of these newer platforms. With competitive costs and the existing scaling, the pooled pyrosequencing approach may be useful in global TDR surveillance through its implementation at specialized HIV DR laboratories [17].

Although pyrosequencing pooled specimens is a promising approach for TDR surveillance, there are limitations to extrapolating the results from our study. The high sensitivity and data throughput of pyrosequencing result in high number of NT variants being detected, many of which exist at low frequencies. The phylogenetic analysis is helpful in resolving this issue but further improvements will be obtained by using the multiplex identifier approach. Second, in order to accurately reflect the true TDR prevalence in the viral population, the PCR amplicons from each component specimen need to be pooled at equimolar concentrations with extreme precision. Any bias introduced at this step can have a significant effect on reported TDR prevalence. Third, although high numbers of sequence reads are generated for each pyrosequencing run, the quality and usability of these reads vary. Stringent sequence alignment criteria are essential to screen out unreliable shorter reads and those containing false insertion/deletion mutations.

In summary, we have demonstrated that pooled pyrosequencing of the HIV protease identifies protease TDR mutations with comparable costs to conventional Sanger sequencing. Pyrosequencing results were highly correlated with those produced by conventional Sanger sequencing of individual specimens. Advances in labeling of pyrosequencing specimens will facilitate linkage TDR mutations across the *pol* gene making it possible to use this approach for TDR surveillance for mutations in both protease and reverse transcriptase. The significance of minor sequence variants, which exist within individuals in surveillance cohort but below the detection threshold of conventional techniques, remains to be explored. Applications for this technique may be found in other disciplines where surveillance of mutations within a population is required.

## Supporting Information

Table S1 Comparison of material costs for DR testing of 96 specimens.
(0.05 MB DOC)

Table S2 Comparison of labour cost for DR testing of 96 specimens.
(0.05 MB DOC)

Table S3 Comparison of total cost for DR testing of 96 specimens.
(0.03 MB DOC)
