# Supplementary material for: HIV Drug Resistance Surveillance Using Pooled Pyrosequencing
Source: PLoS One. 2010 Feb 17;5(2):e9263. doi: 10.1371/journal.pone.0009263 (PMC2822863; doi:10.1371/journal.pone.0009263)
Supplement: Table S1 — Comparison of material costs for DR testing of 96 specimens. (0.05 MB DOC) [file pone.0009263.s001.doc]

**Table S1 Comparison of material costs for DR testing of 96 specimens.**

|  | **Cost in CDN$** | | | |
| --- | --- | --- | --- | --- |
|  | **Sanger sequencing**  **(PR only)** | **Sanger sequencing**  **(PR+RT)** | **Pyro-sequencing**  **(PR only)** | **Pyro-sequencing**  **(PR +RT)*** |
| **RNA extraction**  BioMerieux EasyMag | 643 | 643 | 643 | 643 |
| **PCR / Sequencing** |  |  |  |  |
| RT-PCR | 743 | 743 | 743 | 743 |
| ***** Nested-PCR | 311 | 311 | 311 | 933 |
| ***** Gel Electrophoresis | 87 | 87 | 87 | 261 |
| ***** PCR Clean-up | 97 | 97 | 97 | 291 |
| BigDye terminator | 2,243 | 4,486 | NA | NA |
| Clean-up: Sephadex | 111 | 222 | NA | NA |
| Other sequencing PCR  and Capillary sequencing supplies | 364 | 728 | NA | NA |
| DNA quantification / pooling | NA | NA | 29 | 87 |
| Library prep | NA | NA | 177 | 177 |
| **†** emPCR | NA | NA | 194 | 388 |
| ***** Pyrosequencing | NA | NA | 222 | 666 |
|  |  |  |  |  |
| **Total cost** | $4,599 | $7,317 | $2,503 | $4,189 |
|  |  |  |  |  |
| **Material cost/specimen** | $47.90 | $76.22 | $26.07 | $43.64 |

Notes:

***** Costs for amplification, purification, quantification and pyrosequencing of both protease and RT will be 3x the cost of performing the same steps on protease alone, as analysis would be done on 3 x 400bp fragments.

**†**Cost for emPCR is only doubled for pyrosequencing as only two of the larger scale reactions are required to obtain sufficient oversampling. For more details please see supporting itemized table.
