# Supplementary material for: HIV Drug Resistance Surveillance Using Pooled Pyrosequencing
Source: PLoS One. 2010 Feb 17;5(2):e9263. doi: 10.1371/journal.pone.0009263 (PMC2822863; doi:10.1371/journal.pone.0009263)
Supplement: Table S2 — Comparison of labour cost for DR testing of 96 specimens. (0.05 MB DOC) [file pone.0009263.s002.doc]

**Table S2** **Comparison of labour cost for DR testing of 96 specimens.**

|  | **Time in Hours** | | | |
| --- | --- | --- | --- | --- |
|  | **Sanger sequencing**  **(PR only)** | **Sanger sequencing**  **(PR+RT)** | **Pyro-sequencing**  **(PR only)** | **Pyro-sequencing**  **(PR+RT)** |
| **RNA extraction**  BioMerieux EasyMag | 2 | 2 | 2 | 2 |
| **PCR / Sequencing** |  |  |  |  |
| RT-PCR set-up | 1.5 | 1.5 | 1.5 | 1.5 |
| Nested-PCR set-up | 1.5 | 1.5 | 1.5 | 2.5 |
| Gel Electrophoresis | 1 | 1 | 1 | 2 |
| PCR Clean-up | 1.5 | 1.5 | 1.5 | 2.5 |
| BigDye Reaction | 2 | 4 | NA | NA |
| Sequencing Clean-up | 1 | 1.5 | NA | NA |
| Capillary sequencing | 1.5 | 3 | NA | NA |
| DNA quantification / pooling † | NA | NA | 0.5 | 1 |
| Library Prep | NA | NA | 0.5 | 1 |
| emPCR | NA | NA | 5.5 | 11 |
| Pyrosequencing‡ | NA | NA | 3 | 4 |
| **Data Analysis** | 4 | 7.5 | 7.5 | 7.5 |
|  |  |  |  |  |
|  |  |  |  |  |
| **Total Hands-on time** | 16.0 | 23.5 | 24.5 | 35 |
| **Total labour cost with technician cost @ $25/hr** | $400.00 | $588.00 | $613.00 | $875.00 |
| **Labour cost / specimen** | $4.17 | $6.13 | $6.39 | $9.11 |

Notes:

† This step is automated using a robotic system.

‡ Scaling of pyrosequencing set-up is non-linear.
