# Supplementary material for: HIV Drug Resistance Surveillance Using Pooled Pyrosequencing
Source: PLoS One. 2010 Feb 17;5(2):e9263. doi: 10.1371/journal.pone.0009263 (PMC2822863; doi:10.1371/journal.pone.0009263)
Supplement: Table S3 — Comparison of total cost for DR testing of 96 specimens. (0.03 MB DOC) [file pone.0009263.s003.doc]

**Table S3** **Comparison of total cost for DR testing of 96 specimens.**

|  | **Cost in CDN$** | | | |
| --- | --- | --- | --- | --- |
|  | **Sanger sequencing**  **(PR only)** | **Sanger sequencing**  **(PR+RT)** | **Pyro-sequencing**  **(PR only)** | **Pyro-sequencing**  **(PR +RT)*** |
| **Labour cost / specimen** | $4.17 | $6.13 | $6.39 | $9.11 |
| **Material Cost/specimen** | $47.90 | $76.22 | $26.07 | $43.64 |
| **Total cost/specimen** | $52.07 | $82.35 | $32.46 | $52.75 |
